# Supplementary material for: Feature selection methods affect the performance of scRNA-seq data integration and querying
Source: Nat Methods. 2025 Mar 13;22(4):834–44. doi: 10.1038/s41592-025-02624-3 (PMC11978513; doi:10.1038/s41592-025-02624-3)
Supplement: Supplementary file 1 — Supplementary Methods. [file 41592_2025_2624_MOESM1_ESM.pdf]

nature methods

Supplementary information https://doi.org/10.1038/s41592-025-02624-3

# Feature selection methods affect the performance of scRNA-seq data integration and querying

In the format provided by the authors and unedited

# Supplementary methods

## Benchmark overview

The design of our study is similar to that of existing benchmarks, consisting of a set of test datasets, the feature selection methods to be evaluated and various metrics for measuring performance. Each dataset was processed to ensure a standard input format for the pipeline and split into reference and query batch sets. The feature selection methods were then applied to the reference batches to generate a set of selected features for each method that was used to integrate the reference batches. The query batches were mapped to the integrated reference using the same selected features. A cell label classifier trained on the integrated reference embedding was then used to transfer labels to the query cells. Metrics were calculated at different stages depending on the required inputs. Once all metrics scores were available for all datasets, they were scaled and aggregated before calculating final rankings. The complete benchmarking pipeline is implemented as a Nextflow[1] workflow available from GitHub[2]and archived on Zenodo[3]. Details of the specific methods, metrics, datasets and processing steps are provided in the following sections.

## Evaluated methods

We selected a range of feature selection methods that cover approaches from standard analysis workflows (primarily highly variable gene selection) and alternative methods proposed for scRNA-seq data, in some cases for particular analysis tasks such as clustering. These methods can be divided into categories depending on their underlying methodological approach. Some methods focus on simple statistics of individual features, while others use more complex dataset representations to identify important features. To be considered, a method had to be implemented in a publicly available package that we could reliably install and run based on the provided documentation. Some methods can automatically determine the number of features to select, while this is a user-defined parameter for most others. A few methods can consider batch labels during selection, but for most, this requires manually splitting the data, computing feature sets on each batch and combining the results. For most methods, we have used the default settings or what is recommended in any accompanying documentation, but for a subset of highly-used methods, we evaluated different combinations of parameters to investigate their effect. Any preprocessing steps required before feature selection are considered as part of the method. We used the steps suggested in the documentation for each method as they are recommended by the authors and represent the most likely real-world usage.

### Simple control methods

As controls, we include **all features** and **random** feature sets in the evaluation. These act as points of reference as we want to see when using selected feature sets improves performance over using all features. At the same time, we expect any reasonable feature selection method to outperform randomly selected sets. To control for variability in selecting random features, we always include five random feature sets selected with different seeds and average metric scores over the five sets.

### Excess variability methods

The most commonly used approach to feature selection in standard scRNA-seq analysis toolboxes such as scanpy[4] and Seurat[5] is to select highly variable features or, more specifically, features that show excess

variability beyond what is expected. The assumption for selecting features in this way is that this extra variability results from differences in gene expression between cell populations or states (rather than sample noise) and that selecting these features will identify those important to the cells in the sample.

We benchmarked the following excess variability methods:

- The naive approach to selecting features based on variability is to simply calculate the **variance** of each feature and select those with the highest values. However, this neglects the known relationship between the mean expression of each feature and the variance (mean-variance relationship), where lowly-expressed features are generally more variable due to the greater effect of sampling noise.
- **Brennecke** et al. proposed one of the first feature selection methods for scRNA-seq data that considered the mean-variance relationship[6]. A curve is fitted to the relationship between the normalised mean expression of each feature and the squared coefficient of variation, with the selected features being those that sit significantly above this trend. We use the implementation of this method in the scran package[7] (v1.26.0).
- The **Seurat** package (v4.3.0) contains three excess variability feature selection methods:
    - The simplest is to select features with the highest calculated **dispersion**.
    - The **MVP** (mean.var.plot**)** method (default in older versions of Seurat) bins features based on their mean expression and calculates z-scores for the dispersion of the features within each bin[5]. Features are then selected based on a threshold of standard deviations from the mean dispersion within a bin.
    - The **VST** (variance stabilising transformation) method (current default) fits a LOESS line to the relationship between (log) mean and (log) variance for each gene[8]. This fit is then used to standardise the feature values and the features with the highest standardised variances are selected.
- The **scanpy** package[4] (v1.9.1) provides Python implementations of three highly variable feature selection methods originally developed in other tools. We include these for comparison as they are commonly used and the scanpy workflow suggests different preprocessing and normalisation steps that may affect the selected features. There may also be implementation differences. The scanpy functions can select batch-aware features by performing feature selection per batch and keeping those selected for the most batches (up to the chosen number of features), with ties being broken by the test statistic used.
    - The **Seurat** method implements the Seurat MVP method
    - The **SeuratV3** method implements the Seurat VST method
    - The **CellRanger** method is similar to the Seurat method (Seurat MVP), but features within each bin are normalised using the median and median absolute deviation rather than mean and standard deviation[9]
- The **OSCA** method follows the feature selection approach for batch integration suggested by the "Orchestrating Single-Cell Analysis with Bioconductor" online book[10]. This process involves performing a batch-aware scaling normalisation using the batchelor package[11] (v1.14.0) and modelling the mean-variance relationship with scran by including batch as a block variable. The variance fitted by the trend is assumed to be technical, with residual variance considered the biological component. Fitting is performed per batch and p-values are combined to select features across batches.

## Methods based on other statistical features

Besides variability, other feature statistics can also be used for feature selection:

- A simple, naive method is to select the features with the highest **mean** expression values
- The **Anticor** method (v0.1.8) selects features with excess negative correlations with other features using a permutation-based approach[12]. The intuition behind this method is that genes essential to a cell type's identity will be expressed in some cell types and not others and, therefore, be negatively correlated with marker genes for other cell types. By default, the Anticor method excludes features from a set of predefined gene pathways, but we have disabled that functionality here as it requires that specific gene identifiers be used, which was not the case for all the datasets tested.
- The **NBumi** method from the M3Drop package (v1.24.0) also tries to detect marker genes, but rather than using negative correlations, it looks for features that have an excess of zero counts for the mean expression level[13]. The idea is similar in that features important for identifying a cell type should be highly expressed in that type but not in others. We use the NBumi method instead of the original M3Drop method as it is designed for data collected using unique molecular identifiers (UMIs), while M3Drop instead assumes a distribution appropriate for protocols that cover the full length of RNA transcripts. Here, we select features with an adjusted p-value below 0.01 unless this results in fewer than 500 selected features, in which case the 500 lowest p-value features were used.
- **DUBStepR** (Determining the Underlying Basis using Stepwise Regression) (commit 76aa3948) also considers correlations but uses several steps to select features[14]. First, lowly expressed features, mitochondrial genes, ribosomal genes, and pseudogenes are removed (based on matching gene symbols). A correlation matrix between features is then calculated and features are binned based on mean expression levels. Within each bin, correlations are scaled using an adjusted range and those with low scaled-correlation values are removed. A stepwise regression is then performed where, at each step, the feature explaining the most variance in the correlation matrix is removed. After the regression, the elbow point on a scree plot is used to select an initial set of seed features. The seed feature set is expanded by adding features with the highest Pearson correlation to any seed feature. The final number of selected features is determined by a density index that considers neighbourhoods in PCA (principal component analysis) space.

## Model-based methods

Model-based methods fit an appropriate distributional model to the dataset, typically using the raw counts rather than a normalised and transformed version of the data. Features are then selected by looking for those significantly different from the fitted model.

- The **scTransform** method uses regularised negative binomial regression to normalise UMI count data[15]. Features can be selected by ranking by residual variance from the fitted model. We access scTransform (v0.3.5) via the function in the Seurat package.
- Analytic **Pearson** residuals have also been proposed as a method for normalising UMI data[16]. This approach is similar to scTransform but simplifies the model to an offset model with a set overdispersion. After fitting the model, features can again be selected based on residual variance. We used the implementation of this method in the scanpy package.
- The **scry** method (v1.10.0) fits a multinomial count model to the data[17]. Selected features show high residual deviance from the null model which assumes constant expression across all cells.

## Embedding-based methods

Embedding (or dimensionality reduction) is a commonly used preprocessing step in scRNA-seq analysis to remove noise and reduce size and computational complexity. Some feature selection methods either use sophisticated dimensionality reduction methods that include ranking or weighting of features, or look for features that vary across an embedding rather than in the original feature space.

- **scPNMF** (single-cell Projective Non-negative Matrix Factorization) (commit 47d5b10c) performs a modified PNMF where an alternative initialisation is used[18]. Informative bases are then selected by choosing those not correlated with library size (total counts per cell) and that show a multimodal distribution. Features are selected based on their maximum weighting for the selected bases.
- The **singleCellHaystack** (v0.3.4) method uses Kullback-Leibler divergence to find features expressed in subsets of non-randomly positioned cells in a reduced dimensional space[19]. To generate the embedding space, we first select features using the default Seurat highly variable feature method and then perform a 50-dimensional PCA using these features. singleCellHaystack is then applied to all features using the position of cells in the PCA space.

## Graph-based methods

Another common step in scRNA-seq analysis is to build a nearest-neighbour graph of cells, typically using positions in an embedded space. These graphs can also be used as a starting point for feature selection.

- **Hotspot** (v1.0.0) looks for features with high local auto-correlation within a neighbourhood graph[20]. The graph can be constructed in various ways depending on the use case, but we use a PCA embedding based on all features as input here. Features are first filtered to keep those with a false discovery rate adjusted p-value below 0.05 and then those with the highest test statistic values are selected.
- **triku** (v2.1.4) uses a neighbourhood graph to distinguish features expressed in a few cells randomly across a dataset from those expressed in a few related cells[21]. Expression is aggregated across the neighbourhood of each cell and the distribution is compared to a null distribution of randomly selected cells using the Wasserstein distance. Features with the greatest distance from the null distribution are selected. The neighbourhood graph is constructed in PCA space based on all features, and the number of selected features is automatically decided by triku.

## Supervised methods

In this benchmark, we focus on evaluating unsupervised feature selection methods, as cell labels are typically not available before the integration process we are interested in assessing. However, at least some level of cell labels may be available for some analyses, particularly atlas-building projects that combine previously annotated public datasets. As a baseline supervised method, we include marker genes selected using the **Wilcoxon** rank sum test (as implemented in the scanpy package) followed by a filtering procedure. The test compares the cells for one label against all other cells. The results are then filtered to remove features expressed in less than 10 per cent of cells within that label, expressed in more than 80 per cent of cells outside the label or with a p-value above 0.1. Next, the remaining features are sorted by estimated log-fold change and the top 200 features are selected. The final feature set is the intersection of the features selected for each label.

In addition, we include a supervised method based on known transcription factors. We downloaded a database of human transcription factors from The Human Transcription Factors[22] website[23] and selected

1639 genes where the “Is TF?” field was equal to “Yes”. The intersection of this list with the genes in each dataset is then used for evaluation. This method cannot be applied to the splat dataset due to a lack of correspondence between simulated genes and real transcription factors.

### Stable expression methods

The opposite of selecting highly variable features is selecting those stably expressed or varying less than expected across the dataset. The **scSEGIndex** method implemented in the scMerge package (v1.1.4.0) calculates a feature stability index by fitting a gamma-Gaussian mixture model to each feature[24]. The parameters of this model and other features, such as the proportion of zero counts, are used to rank features and calculate the final stability index. Features selected in this way can be used as alternatives to housekeeping genes for identifying essential functions within a cell but also as negative controls for normalisation or batch correction methods. We used them similarly here and expected that the features selected by scSEGIndex should perform poorly for integration as they should not capture either technical noise between samples or biological signals between cells.

## Evaluation metrics

We implemented a wide array of metrics designed to evaluate different aspects of integrating scRNA-seq samples to create a reference as well as using it by mapping query samples. Some metrics require a ground truth cell label, while others are unsupervised and measure whether the structure in a single sample is maintained after integration or mapping. All metrics are designed so that a raw score of 0 represents the worst possible performance and a raw score of 1 the best possible performance.

### Integration (Batch)

The Integration (Batch) metrics measure the mixing between batches in the reference. In a well-integrated dataset, cells of the same cell type should be thoroughly mixed and neighbourhoods should be equally likely to contain cells from any batch.

- **Batch ASW** (batch Average Silhouette Width) is an adjusted version of Label ASW (see below) that uses batch labels rather than cell labels[25]. Because we want to measure mixing between batches (rather than the usual use case for ASW of evaluating separation between clusters), the scores are adjusted so that a raw silhouette score of 0 (which indicates mixing between labels) gives the highest Batch ASW score. We used the Batch ASW function in the scIB package[25] (v1.1.4), which uses scikit-learn[26] (v1.1.2) to calculate the silhouette scores.
- **Batch PCR** (batch Principal Component Regression) measures how much of the variance in a dataset can be explained by batch labels[25]. This is done by performing a PCA followed by linear regression against the batch labels with the estimated coefficients used to weight the variance explained by each component. The final score is calculated by taking the difference in this value before and after integration. We used the implementation in the scIB package.
- **Graph connectivity** measures the proportion of cells for each cell label that are connected after subsetting a neighbourhood graph to only that label[25]. The score is then averaged across the different labels. This metric is implemented in the scIB package.
- **iLISI** (integration Local Inverse Simpson’s Index) measures the effective number of batches in a neighbourhood by counting how many cells can be drawn until a batch label is encountered twice[27]. We used the graph-based implementation in the scIB package.

- **kBET** (k-nearest neighbour Batch Effect Test) uses a statistical test to evaluate whether the batch composition of the neighbourhood of a cell is similar to the expected global batch composition[28]. It is applied to each label and the resulting scores are aggregated, with the score for each label being the rejection rate of a set of randomly selected cells. We used the function in the scIB package, which wraps the R kBET package (commit a10ffeaa).
- The **mixing** metric measures mixing between batches in the neighbourhood of each cell[29]. For each batch, it identifies the $k^{th}$ nearest neighbour (we used the default value of k = 5) and its rank within the total neighbourhood of the cell. The cell score is then the median across all batches. To calculate a dataset-level score, we divided the cell scores by the maximum possible neighbourhood size, took the mean across all cells and subtracted from 1 (so that higher scores are better). Cell level scores are calculated using the Seurat package.
- **CMS** (Cell-specific Mixing Score) tests for batch effects within the neighbourhood of each cell[30]. It uses the Anderson-Darling test to evaluate if the distributions of distances of cells within the neighbourhood, but from different batches, come from the same distribution. This gives the probability of the data coming from an equally mixed neighbourhood. As a final dataset-level score, we used 1 minus the proportion of cells with a CMS p-value of less than 0.1. The CMS score is implemented in the CellMixS package (v1.14.0).

## Integration (Bio)

The Integration (Bio) metrics measure whether biological signals (primarily cell labels) from individual batches are conserved after integration. Unlike the batch correction metrics, where perfect scores could be obtained by mapping all cells to a single point, the biological conservation metrics require that cell labels are separated in the integrated space.

- **Label ASW** (label Average Silhouette Width) measures the relationship between distances between cells within a cluster and distances between cells in that cluster and other clusters, where clusters correspond to cell labels[25]. The distances are calculated in the integrated embedding space, and the average silhouette width across all labels is used as the final score, which is rescaled between 0 and 1 (from the native range of -1 to 1). This metric was calculated using the function in the scIB package, which uses the scikit-learn implementation of ASW.
- The **local structure** metric calculates the overlap between cell neighbourhoods in a batch-specific PCA space and the integrated embedding[29]. The final score is the average across all cells. This metric was calculated using the Seurat package.
- **cLISI** (cell-type Local Inverse Simpson's Index) is similar to iLISI but measures the number of cells in a neighbourhood that can be drawn until the same cell label is observed twice[27]. We used the graph-based implementation in the scIB package.
- The **cell cycle** conservation score uses principal component regression to assess how much of the variance in the dataset can be attributed to the cell cycle and is preserved after integration[25]. Each cell is scored for the S and G2M phases of the cell cycle using the gene sets from Tirosh et al.[31] and the scoring method suggested by Satija et al.[5] as implemented in scanpy. The gene sets were obtained from the scIB GitHub repository and ENSEMBL gene IDs were added from BioMart[32] using the biomaRt package[33] (v2.54.0). When necessary, these were used instead of the gene symbols in the original files. The difference in the variance associated with the cell cycle scores in each batch compared to the integrated embedding is used to calculate the final score (normalised by the unintegrated variance). We used the implementation in the scIB package. This metric cannot be computed for simulated datasets as they do not include cell cycle effects.

- **ARI** (Adjusted Rand Index) measures the overlap between two sets of clustering labels, in our case, the ground truth cell labels and a set of cluster labels. To generate the clustering assignments, we performed Leiden clustering with the resolution parameter set to values between 0.1 and 2 in steps of 0.1[25]. The selected clustering is that which produced the best ARI score. Both the clustering optimisation and the ARI calculations were performed using functions in the scIB package, which rely on the scikit-learn implementation of ARI.
- **bARI** (balanced Adjusted Rand Index) modifies the ARI metric to account for differences in batch sizes and imbalances between cell labels in different batches[34]. This is done by weighting the ground truth subsets so that they contribute equally to the final score. We used the implementation in the balanced_clustering package[34] (commit a2ae3a4d).
- **NMI** (Normalised Mutual Information) also measures the overlap between two clusterings (similar to ARI) and is calculated between the ground truth cell labels and an optimised Leiden clustering[25]. We used the function in the scIB package, which uses the scikit-learn implementation of NMI.
- **bNMI** (balanced NMI) applies the same adjustment as bARI to the NMI score[34].
- The isolated labels metrics look specifically at the labels present in the fewest batches in each dataset[25]. Two different scores are calculated using the scIB package:
  - **Isolated labels ASW** calculates the Average Silhouette Width between the isolated label and non-isolated label cells. This is done for each isolated label and the average is taken as the final score.
  - **Isolated label F1** optimises Leiden clustering to find the clustering that gives the highest F1 score between the isolated label and other labels. This is repeated for each isolated label, and the average is calculated.
- **ldfDiff** (local density factor difference) measures changes in the relative density of the neighbourhood around each cell following integration[30]. The local density factor[35] is calculated for each cell in a batch-specific PCA space and in the integrated embedding, and the difference between them is calculated. To get a cell-level score, we took the absolute value of the distance and set an upper bound of 1 (any values above this are set to 1). The final score is 1 minus the mean cell score. Cell-level ldfDiff scores were calculated using the CellMixS package.

## Mapping quality

The mapping quality metrics assess how well the reference represents the query and is able to merge it into the same space. For a perfect mapping, cell types present in both the reference and query should be mixed, as should batches within the query, while at the same time, biology within the query should be preserved.

- The **cell distance** metric calculates the Mahalonobis distance between each mapped query cell and the distribution of the corresponding label in the reference[36]. The intuition behind this metric is that each cell should be mapped close to (or overlap) the matching cell population in the reference. The Mahalonobis distance is naturally unbounded, so we use an internal boundary by calculating the distance for every cell in the reference for a label and taking the 90$^{th}$ quantile. Query cells that are further than this distance for the corresponding label are considered to be poorly mapped. The final score is 1 minus the proportion of cells outside the boundary.
- The **label distance** is similar to the cell distance but considers labels as a whole rather than individual cells[36]. The Mahalonobis distance is calculated between the centroid of the label in the query and that label in the reference. Labels are skipped if they have fewer than 20 cells in the query or are not in the reference. As with the cell distance, the range is naturally unbounded, so to create a boundary, we used a limit of the maximum distance of query cells to their label centroid.

Distances to the matching reference label are then scaled using this value and set to 1 if they exceed the maximum distance. The final score is the mean across cell types.

- **mLISI** (mapping Local Inverse Simpson's Index) is the same as iLISI but measures mixing between the query and reference rather than batches (also known as ref_query LISI[36]).
- **qLISI** (query Local Inverse Simpson's Index) is the same as iLISI but measures mixing between query batches after mapping to the reference (also known as query_donors LISI[36]).
- **kNN-corr** (k-Nearest Neighbour Correlation) measures how well the neighbourhood of cells in the query is maintained[36]. For each query batch, a PCA is performed and the Euclidean distances to the 100 nearest neighbours of each cell are calculated. The distances to the same neighbours in the joint integrated embedding are also calculated and the Spearman correlation between the two sets of distances is computed. After adjusting the correlations to the range 0 to 1, the mean of cells in each batch is calculated and the final score is the mean across batches. For particularly bad integrations (i.e. small random feature sets), a cell may be equally distant from all neighbours, in which case the correlation can not be calculated and it is assigned a score of 0.
- The **reconstruction** metric was recently proposed for assessing mapping performance using generative deep learning integration methods (such as scVI and scANVI)[37]. It assesses the model's ability to represent query cells by sampling from the posterior distribution for each query cell and measuring the cosine distance between the mean posterior expression profile and the true cell expression profile. We adjusted the distances to be in the range 0 to 1 and took 1 minus the mean distance as the final score. This metric cannot be calculated for Symphony integrations as it is not a generative method.

## Label transfer

The label transfer (or classification) metrics measure how well a classifier trained on the reference can correctly predict labels for query cells. This is a classic classification problem, and as such, we use standard classification metrics.

- **Accuracy** measures the proportion of observations in the query dataset that were assigned the correct label. We use the scikit-learn implementation.
- **F1 score** is the harmonic mean of precision (or true positive rate, the proportion of identified labels that were truly positive) and recall (the proportion of true labels that were identified). As it assumes binary classification, it is calculated separately for each label and then averaged across labels in different ways to give a final score.
    - Macro averaging is the simple mean of the per-label scores without any weighting. This is implemented directly in the scikit-learn package.
    - Micro averaging is the mean across cells. It is implemented in the scikit-learn package and is equivalent to accuracy.
    - To place more emphasis on rarer cell labels, we also use a rarity-weighted averaging. Here, the weight for each label is the inverse of the frequency of that label times the sum of the inverse frequencies of all labels[38].
- The **Jaccard Index** compares two sets by dividing the size of the intersection by the size of the union. It can be used to evaluate classification by considering the set of observations predicted to have a label with the set of observations with that ground truth label. As with the F1 score, it is calculated for each label and then averaged (using functions in the scikit-learn package).
- **MCC** (Matthews Correlation Coefficient) is a balanced classification metric that considers all combinations of true and false, positive and negative outcomes[39] (also known as the Yule Phi

coefficient[40]). As it is a correlation, the native range is -1 to 1, which we convert to 0 to 1 to get the final score. MCC was calculated using the scikit-learn package.
- **AUPRC** (Area Under the Precision-Recall Curve) considers class probabilities rather than just the assigned labels and is conceptually equivalent to calculating the average precision over many different probability cutoffs. We used the scikit-learn average precision score function which uses the increase in recall from the previous threshold to weight the precision. The AUPRC score is calculated per label and macro-averaged.

### Unseen population prediction

The final category of metrics focuses on novel biology in the query. It aims to measure how mapping has affected cell labels present in the query but deliberately left out of the reference. In an ideal mapping, these should be maintained as separate populations. However, an integration that does not properly understand the variation in the unseen population may merge it with another label.

- The **unseen uncertainty** metric uses the output of the label transfer classifier. If unseen populations in the query are separated from the reference, we expect the classifier to be less confident in assigning them a label. Based on this assumption, the uncertainty metric is calculated as 1 minus the mean probability of the assigned class for query cells from unseen populations.
- The **unseen cell distance** is based on the cell distance mapping metric but calculated only for unseen query populations. As the label does not exist in the reference, we calculate distances to each cell's nearest reference population. We also subtract the final score from 1 so that higher distances (greater separation from the reference) give higher scores.
- The **unseen label distance** is based on the label distance mapping metric with similar changes to the unseen cell distance metric. Distances are calculated to the nearest reference label, and scores are adjusted so that higher scores represent greater separation from the reference.
- **Milo** is a method for identifying cell neighbourhoods that show differential abundance associated with a covariate[41]. It was recently used as a metric for identifying previously unseen populations in a benchmark of different experimental designs for integration[37]. Following the example from this benchmark, a neighbourhood graph is calculated in the combined query and reference integrated embedding using a number of neighbours equal to five times the number of batches (up to a maximum of 200). Milo is then applied to a subset of cells (up to 20,000 cells or 10 per cent of the datasets, whichever is higher), with a label specifying whether each cell is a member of the query or reference as the covariate of interest. The results of the Milo test indicate whether query cells are enriched in a cell neighbourhood. We consider this a positive result for unseen cells as variation present in only the query has been conserved. The individual test results are summarised for each unseen label by taking the proportion of cell neighbourhoods significantly associated with the query (FDR adjusted p-value less than 0.1). The final overall score is the average of the proportions across all unseen labels. In rare cases for poor integrations where Milo cannot select cells from an unseen label, that label is assigned a score of 0. We used the implementation in the milopy package (commit be1a6cc8).

## Benchmarking datasets

We selected the datasets in this evaluation to represent a range of scenarios in which integration is a critical analysis step, including smaller-scale datasets and larger atlas-building efforts. They cover a range of tissues, technologies and developmental stages. We chose query batches by selecting a set of batches with shared characteristics different from the remaining reference samples, such as technology, timepoint or location.

The unseen populations only present in the query were chosen by first looking for labels enriched in the query batches and then selecting labels presenting different challenges, such as rare or perturbed cells. For each dataset, we use the cell labels assigned by the original authors with only minimal further quality control applied, as described in the pipeline section below. Any further dataset-specific processing and details, such as the batch and label variables used, are described in the following sections.

## scIB Pancreas

Datasets containing single cells from pancreas samples captured using different technologies are the most commonly used example for evaluating scRNA-seq integration methods. We used the version prepared for the scIB project[25] and downloaded it from figshare[42]. Cell labels were taken from the “celltype” cell annotation column (12 reference labels) and batches from the “tech” column. For the query, we used the batches representing the CEL-seq and CEL-seq2 technologies with the “activated_stellate” label treated as an unseen population only present in the query. After preparation, the dataset contained 18319 features, 12731 reference cells (7 batches) and 3243 query cells (2 batches).

## NeurIPS 2021

The NeurIPS dataset was created for the 2021 NeurIPS Multimodal Single-Cell Data Integration competition[43]. The dataset consists of bone marrow mononuclear cells collected from several donors and sequenced across multiple sites, with some donors being sequenced multiple times[44]. Different technologies were used to measure RNA and protein (CITE-seq) or RNA and chromatin accessibility (10x Multiome). We downloaded the CITE-seq dataset from the Gene Expression Omnibus (GEO) database[45] (GSE194122) and used only the gene expression features. Cell labels were taken from the “cell_type” annotation and batch labels from the “batch” annotation. We considered samples from Site 4 as the query with the “CD8+ T naive” and “Proerythroblast” treated as unseen query populations. After preparation, the dataset contained 13953 features, 70061 reference cells (9 batches) with 42 reference labels and 16715 query cells (3 batches).

## Fetal liver hematopoiesis

The fetal liver hematopoiesis dataset from Popescu et al.[46] was used to evaluate the Symphony query mapping method[36]. This dataset catalogues the cells in the hematopoietic liver during human development. We downloaded the data provided by the original authors from CellAtlas.io[47]and used batch labels from the “fetal.ids” annotation and cell labels from the “cell.ids” annotation. Three samples from different developmental stages were treated as the query with "Kupffer Cell”, “NK”, “ILC precursor” and “Early lymphoid_T lymphocyte" labels present in the query but excluded from the reference. The prepared dataset contains 26686 features, 62384 reference cells (11 batches, 23 reference labels) and 26449 query cells (3 batches).

## Reed Breast

The Reed breast dataset is a recently released atlas that profiles cells from the breast tissue of 55 healthy women with different characteristics, including age, ethnicity, sampling location and known genetic risk factors[48]. We downloaded the dataset released with the preprint[49] from the CELLxGENE Data Portal[50] (Dataset ID: 0ba636a1-4754-4786-a8be-7ab3cf760fd6, Census version: 2023-07-05) using the cellxgene-census package (v1.0.1) and subsetted to cells with a BRCA status of either wildtype (“WT” or “assumed_WT”) or “BRCA1”. Donor ID was used as the batch label, with cell labels taken from the “level2”

annotation. We also excluded a subset of cells labelled as doublets, as it is not clear how the different metrics should consider them. Wildtype cells were used to create the reference, and BRCA1 cells were used as the query. The “BSL2”, “CD8T 1”, “CD8T 2”, “CD8T 3”, “FB5”, “LEC1” and “LEC2” labels were used as unseen labels present only in the query. After preparation, the dataset contained 33691 features, 337339 reference cells (24 batches, 32 reference labels) and 197649 query cells (17 batches).

## single cell Eye in a Disk (scEiaD)

The single cell Eye in a Disk (scEiaD) dataset was constructed by integrating publicly available ocular datasets from three species (human, mouse and macaque)[51]. We downloaded the dataset from the PLatform for Analysis of scEiad website[52] and selected only the human cells derived from tissue samples where the organ was specified as “Eye”. We also removed cells that did not have a cell label or were labelled as doublets and batches with fewer than 500 cells remaining, as we found these caused some metrics to produce unreliable results. Cell labels were taken from the “CellType_predict” annotation (the result of a classifier trained by the authors to predict and harmonise labels following integration), and the defined “batch” annotation was used for batches. We split batches using the cell capture technology, with 10x version 2 taken as the reference and 10x version 3 and Drop-seq batches making up the query. The “B-Cell”, “Blood Vessel”, “Macrophage”, “Pericyte”, “Smooth Muscle Cell” and “T/NK-Cell” labels are only present in the query. After preparation, the dataset contained 19560 features, 360270 reference cells (69 batches, 41 reference labels) and 48496 query cells (18 batches).

## Human endoderm

The human endoderm dataset combines human foetal samples to profile the development of multiple endoderm-derived organs[53]. We downloaded the dataset from the Mendeley Data repository provided by the authors[54]. Individuals were treated as batches with labels obtained from the “Cell_type” annotation, and a small number of cells labelled as “Undefined” were removed. Samples from weeks 12-15 of the developmental trajectory were selected as the query with "Basal like”, “Ciliated”, “Hepatocyte”, “Mesenchyme subtype 4” and “T cell/NK cell 1" labels treated as query-specific. The prepared dataset consisted of 27855 features, 100580 reference cells (10 batches, 21 reference labels) and 44784 query cells (4 batches).

## Human Lung Cell Atlas (HLCA)

The Human Lung Cell Atlas (HLCA) represents a comprehensive effort to catalogue the diversity of cell types in the healthy and diseased lung by combining publicly available and newly produced datasets[55]. The core atlas combines samples from several datasets, including different sampling locations and techniques, various sample preparation technologies and protocols, and individuals diverse in factors such as age, sex, location, ethnicity and smoking status. Following integration, the authors produced a detailed consensus annotation at several resolution levels. The samples' diversity and the labels' specificity mean this dataset represents perhaps the most significant integration challenge of those selected for this study. We downloaded the core HLCA dataset from the CELLxGENE Data Portal (Dataset ID: 066943a2-fdac-4b29-b348-40cede398e4e, Census version: 2023-07-25) and used the “dataset” annotation as defined by the authors as batch labels with “ann_finest_level” annotation as labels. The subject type was used to construct the query and reference, with datasets from organ donors treated as the reference and healthy and diseased samples from living donors making up the query. This division is also related to technical covariates such as the tissue sampling method. Several labels are treated as only present in the query, specifically "Multiciliated (nasal)”, “Club (nasal)”, “Goblet (subsegmental)”, “SMG serous (nasal)”,

“SMG serous (bronchial)”, “SMG mucous”, “EC aerocyte capillary”, “Peribronchial fibroblasts”, “Smooth muscle”, “Smooth muscle FAM83D+”, “B cells”, “DC2”, “Alveolar Mph CCL3+” and “Mast cells". After preparation, the final benchmarking dataset includes 27987 features, 314573 reference cells (9 batches, 47 reference labels) and 251400 query cells (5 batches).

### HLCA (immune)

The HLCA (immune) dataset takes the full HLCA dataset and uses the coarsest level of annotation to select cells in the immune compartment. The motivation for including this subset as a separate dataset is to allow some insight into how feature selection and integration perform on a single lineage, as this has been suggested as an alternative approach for analysing large studies of diverse tissues. The batches and labels are the same as the full HLCA dataset, but after subsetting, only "B cells”, “DC2”, “Alveolar Mph CCL3+” and “Mast cells" remain as unseen labels in the query. There are also fewer batches as some did not contain sufficient numbers of immune cells. The HLCA (immune) dataset consists of 26618 features, 155385 reference cells (7 batches, 16 reference labels) and 52795 query cells (2 batches).

### HLCA (epithelial)

The HLCA (epithelial) dataset is a second subset of the HLCA dataset, constructed similarly but focusing on the epithelial compartment. This subset consists of 27673 features, 118374 reference cells (8 batches, 17 reference labels) and 162875 query cells (5 batches) with “Multiciliated (nasal)”, “Club (nasal)”, “Goblet (subsegmental)”, “SMG serous (nasal)”, “SMG serous (bronchial)”, and “SMG mucous” remaining as unseen labels in the query.

### splat

Evaluations using real datasets provide the most accurate assessments of performance, but they also present challenges as they rely on ground truth from previous analyses, which may be incomplete or biased towards the methods that were originally used. Some of these concerns can be addressed by simulations where a definite ground truth is known. We have created a simulated dataset using a modified version of the splat simulation in the Splatter package[56] (v1.25.1). This simulation has been designed to represent a scenario where a tissue is measured using three different technologies (two batches each) in two conditions. These “technologies” measure a medium number of cells at medium depth (Batch1, Batch2), a low number of cells at high depth (Batch3, Batch4) and a high number of cells at low depth (Batch5, Batch6), with the first two comprising the reference and the last one the query. The simulation contains 10 cell labels, including a progenitor differentiating along two trajectories (one with an intermediate cell type only present in the query) and six discrete cell types that differ in number of cells, number of differentially expressed genes and number of detected features. The discrete groups include a rare population and a perturbed state, which are only present in the query. To increase the variability in the simulation, we added additional label-specific noise factors to the model, which were applied just before generating counts. After preparation, the splat dataset contains 9984 features, 30041 reference cells (4 batches, 7 reference labels) and 69936 query cells (2 batches). The “Intermediate”, “Rare” and “Perturbed” labels are only present in the query.

## Benchmarking pipeline

To implement and apply the methods and metrics, we built a pipeline using Nextflow[1]. By using a workflow manager to construct the benchmark, we improve reproducibility, make sure that results are up-to-date as

code is updated and easily take advantage of computing resources. The Nextflow pipeline takes a dataset as input, applies some standard preprocessing and splits it into reference and query samples with annotations stored in standard locations. The feature selection methods are applied to the reference, and each feature set is provided to the integration stage. After integration, the query is mapped to the reference, and a cell label classifier is trained. The reference and query (before and after integration or mapping), ground truth cell labels and transferred labels are provided to metrics as required. The metric scores are then scaled and aggregated. Final method rankings are calculated after considering results from all datasets. Pipeline stages used both Python (v3.9.13) and R[57] (v4.2.2), including packages from Bioconductor[58] The Python anndata package[59] (v0.8.0) was used to store data and save it as H5AD files between pipeline stages. The zellkonverter package (v1.8.0) was used to load data into R via the reticulate (v1.26) interface where it was stored as SingleCellExperiment[10] (v1.20.0) or SeuratObject (v4.1.3) objects.

## Dataset preprocessing

The first stage of the pipeline is to apply standard preprocessing to each dataset. This step includes basic quality control filtering of cells and storing information in locations expected by the pipeline. Cells are labelled with batch and annotation labels, and species information is recorded. We remove cells with fewer than 100 total counts or express fewer than 100 features. The dataset is then split into a reference and query based on the predefined batch labels. Annotation labels with fewer than 20 cells are removed from both the reference and query, as some metrics can behave unpredictably when there are very small cell numbers. Annotations defined as unseen query populations are also removed from the reference. The final preprocessing step removes any features not expressed in any selected cells in the reference. Any further feature selection is performed using the methods to be evaluated.

## Integration and query mapping

The deep learning base model we use for integration is scVI (single-cell Variational Inference)[60], available in the scvi-tools package[61] (v0.17.1). This model is based on a conditional variational autoencoder, which has been shown to perform well in previous benchmarks of integration methods[25]. Importantly for our use, it allows the mapping of query samples using the architecture surgery approach from the scArches package[62]. In this approach, the network weights trained on the reference are frozen, and a small adaptor network is trained for each query batch. We also train a scANVI (single-cell ANnotation using Variational Inference) model[63], a semi-supervised extension of scVI where known cell labels are used to finetune the network. We included scANVI to test whether an integration model with more prior knowledge of the biological signal in a dataset could overcome limitations in a selected feature set. Another advantage of using the scvi-tools models is that they take raw count data as input, which means that we do not have to consider the interaction between feature selection and normalisation methods at the integration stage (a specific normalisation is still used by many of the feature selection methods).

As an example of alternative approaches based on correcting a PCA space, we include integration with the Harmony method[27] followed by query mapping using the associated Symphony approach[36]. This approach represents an alternative class of integration methods and was used to see if the performance of feature selection methods is consistent when compared to deep learning-based integration. As suggested by the documentation, we provide Harmony with normalised expression values rather than raw counts. Counts are first normalised to counts per 10,000, then log-transformed. The dataset is then subset to the provided features and scaled with a maximum value of 10 (per feature) before calculating 30 principal components that are provided as input to Harmony. For query mapping using Symphony, log-transformed normalised query data is provided (scaling is performed as part of the mapping function). Data preprocessing steps are

performed using functions in the scanpy package, and integration and query mapping are performed using the harmonypy[64] (v0.0.9) and symphonypy[65] (v0.2.1) packages.

### Label transfer

To transfer labels from the reference to the query, we trained a multinomial logistic regression classifier on the integrated reference using the scikit-learn package, taking the position of each cell in the integrated embedding space as input and the ground truth cell labels as the output. Labels are transferred to the query by providing the mapped embedding coordinates to the trained classifier, predicting the probability for each reference label and recording the label with the highest prediction probability.

## Metric selection

We used a wide range of metrics for this study, many of which had not previously been rigorously evaluated, particularly for this feature selection task. There are various reasons that a metric may be unsuitable. For example, it may have a small dynamic range, be highly correlated with another metric, perform differently for different integration methods, or be affected by a variable of interest (such as the number of selected features). For that reason, we included a metric selection step. This process consisted of a sweep over different numbers of randomly selected features across all the test datasets. By using random features, we could explore a wide range of possible feature sets while at the same time avoiding biasing metric selection towards any of the real methods. We also included feature sets of different sizes from the scanpy-Seurat method to evaluate the relationship with the number of features as random gene sets have no inherent ordering (i.e. the first features selected are not necessarily more informative than the last features selected). Once we had metric scores for each feature set, we evaluated the behaviour of individual metrics and the relationships between them. Metrics were removed if they could not distinguish between feature sets (have an insufficient dynamic range), were overly correlated (Pearson correlation) with the number of features, were associated with technical dataset features, or showed undesirable correlation patterns. The outcome of the metrics selection process was a non-redundant set of features with fewer biases covering the categories of interest for the final benchmark.

## Selecting a number of features

Most feature selection methods evaluated here require the user to specify the number of features to select. But, while this can affect downstream analyses, there is no clear guidance on how many features should be selected and how that is related to biological factors such as the diversity of cell types in the dataset. To address this question, we evaluated different numbers of features for methods in scanpy and Seurat, as well as features with high variance or high mean expression. Using the scaled values standardised per dataset and method allowed us to see how performance changes with the number of features. While it would be interesting to have this data for all methods, each additional number of features is a significant computational cost. We limited this part of the analysis by methods rather than datasets as it allowed us to see the effect of the number of features across datasets. The results of this analysis informed the number of features we used for most methods in the full evaluation (2000).

## Analysis of results

The absolute values of individual metrics are often of little interest. Instead, the relative performance of methods and the aggregation across metrics are more informative. Despite the raw scores of all of our metrics being adjusted to fall in the range of 0 to 1 (with higher scores indicating better performance), they

continue to have different real dynamic ranges, which can vary across datasets. The first step in aggregating metrics is to scale each metric for each dataset. This scaling can be accomplished in different ways, for example, by scaling between the minimum and maximum values or using the mean and standard deviation. For this study, we used a set of baseline reference methods to establish the range of each metric. These are all features, randomly selected features, stably expressed features selected using the scSEGIndex method and batch-aware features using the Cell Ranger method implemented in the scanpy package. Depending on the metric, using all features performs either well or poorly, while random and stably expressed features result in high batch correction scores but poor conservation of biological information. The scanpy-CellRanger method is included as an example of current standard practice[25,66] and performs well across metrics. The baseline methods were used to establish a reasonable range for each metric (for a dataset), and then all scores were scaled relative to that range. The advantage of using baseline methods rather than scaling across all methods is that the ranges are more interpretable as we know what they correspond to. Adding or removing methods (outside of the baselines) also doesn't affect the reference ranges and scaling.

The scaled metric scores were then aggregated by taking the mean to get an overall score for each category. This aggregation gives a summarised performance for each of the methods for each task. A final overall score for each method is obtained using a weighted mean of the task scores.

$$Overall = \frac{1}{2} * \left(\frac{Int.Batch}{2} + \frac{Int.Bio}{2}\right) + \frac{1}{2} * \left(\frac{Mapping}{3} + \frac{Class.}{3} + \frac{Unseen}{3}\right)$$

This weighting equally balances the reference and query and each metric category. The performance of methods was aggregated and ranked at the level of metric categories, datasets and over the whole benchmark. These rankings let us evaluate which methods perform better at different tasks or scenarios. We also checked for consistency between integration approaches and variants of feature selection methods.

Further analysis examined the similarity between methods by considering the overlap in selected sets using the Jaccard Index. This helped to explain method performance by relating differences in selected features to differences in performance.

Another focus of the analysis was to compare the full HLCA dataset and subsets representing the immune and epithelial compartments. This comparison allowed us to see how feature selection methods perform differently when a dataset is limited to more similar cell types.

Final figures were produced using the ggplot2 package[67] (v3.5.0) and assembled using patchwork (v.1.2.0). Data processing was performed using tidyverse[68] (v2.0.0) packages.

# References


1. Di Tommaso, P., Chatzou, M., Floden, E. W., Barja, P. P., Palumbo, E. & Notredame, C. Nextflow enables reproducible computational workflows. *Nat. Biotechnol.* **35,** 316–319 (2017).

2. Theis lab, @lazappi, @cramsuig, @WWXkenmo, @SabrinaRichter, @amitfrish & @rkrubens. *atlas-feature-selection-benchmark: Code for 'Feature selection methods affect the performance of scRNA-seq data integration and querying'*. (GitHub). at

<https://github.com/theislab/atlas-feature-selection-benchmark>

3. Zappia, L., Richter, S., Ramírez-Suástegui, C., Kfuri-Rubens, R., Weixu, W., Dietrich, O., Frishberg, A., Luecken, M. D. & Theis, F. J. *Code for 'Feature selection methods affect the performance of scRNA-seq data integration and querying'*. (Zenodo, 2024).
4. Wolf, F. A., Angerer, P. & Theis, F. J. SCANPY: large-scale single-cell gene expression data analysis. *Genome Biol.* **19,** 15 (2018).
5. Satija, R., Farrell, J. A., Gennert, D., Schier, A. F. & Regev, A. Spatial reconstruction of single-cell gene expression data. *Nat. Biotechnol.* **33,** 495–502 (2015).
6. Brennecke, P., Anders, S., Kim, J. K., Kołodziejczyk, A. A., Zhang, X., Proserpio, V., Baying, B., Benes, V., Teichmann, S. A., Marioni, J. C. & Heisler, M. G. Accounting for technical noise in single-cell RNA-seq experiments. *Nat. Methods* **10,** 1093–1095 (2013).
7. Lun, A. T. L., McCarthy, D. J. & Marioni, J. C. A step-by-step workflow for low-level analysis of single-cell RNA-seq data. *F1000Res.* **5,** (2016).
8. Stuart, T., Butler, A., Hoffman, P., Hafemeister, C., Papalexi, E., Mauck, W. M., 3rd, Hao, Y., Stoeckius, M., Smibert, P. & Satija, R. Comprehensive Integration of Single-Cell Data. *Cell* **177,** 1888–1902.e21 (2019).
9. Zheng, G. X. Y., Terry, J. M., Belgrader, P., Ryvkin, P., Bent, Z. W., Wilson, R., Ziraldo, S. B., Wheeler, T. D., McDermott, G. P., Zhu, J., Gregory, M. T., Shuga, J., Montesclaros, L., Underwood, J. G., Masquelier, D. A., Nishimura, S. Y., Schnall-Levin, M., Wyatt, P. W., Hindson, C. M., Bharadwaj, R., Wong, A., Ness, K. D., Beppu, L. W., Deeg, H. J., McFarland, C., Loeb, K. R., Valente, W. J., Ericson, N. G., Stevens, E. A., Radich, J. P., Mikkelsen, T. S., Hindson, B. J. & Bielas, J. H. Massively parallel digital transcriptional profiling of single cells. *Nat. Commun.* **8,** 14049 (2017).
10. Amezquita, R. A., Lun, A. T. L., Becht, E., Carey, V. J., Carpp, L. N., Geistlinger, L., Martini, F., Rue-Albrecht, K., Risso, D., Soneson, C., Waldron, L., Pagès, H., Smith, M. L., Huber, W., Morgan, M., Gottardo, R. & Hicks, S. C. Orchestrating single-cell analysis with Bioconductor. *Nat. Methods* 1–9 (2019). doi:10.1038/s41592-019-0654-x
11. Haghverdi, L., Lun, A. T. L., Morgan, M. D. & Marioni, J. C. Batch effects in single-cell RNA-sequencing data are corrected by matching mutual nearest neighbors. *Nat. Biotechnol.* (2018).

doi:10.1038/nbt.4091

12. Tyler, S. R., Lozano-Ojalvo, D., Guccione, E. & Schadt, E. E. Anti-correlated feature selection prevents false discovery of subpopulations in scRNAseq. *Nat. Commun.* **15,** 699 (2024).
13. Andrews, T. S. & Hemberg, M. M3Drop: Dropout-based feature selection for scRNASeq. *Bioinformatics* (2018). doi:10.1093/bioinformatics/bty1044
14. Ranjan, B., Sun, W., Park, J., Mishra, K., Schmidt, F., Xie, R., Alipour, F., Singhal, V., Joanito, I., Honardoost, M. A., Yong, J. M. Y., Koh, E. T., Leong, K. P., Rayan, N. A., Lim, M. G. L. & Prabhakar, S. DUBStepR is a scalable correlation-based feature selection method for accurately clustering single-cell data. *Nat. Commun.* **12,** 5849 (2021).
15. Hafemeister, C. & Satija, R. Normalization and variance stabilization of single-cell RNA-seq data using regularized negative binomial regression. *Genome Biol.* **20,** 296 (2019).
16. Lause, J., Berens, P. & Kobak, D. Analytic Pearson residuals for normalization of single-cell RNA-seq UMI data. *Genome Biol.* **22,** 258 (2021).
17. Townes, F. W., Hicks, S. C., Aryee, M. J. & Irizarry, R. A. Feature selection and dimension reduction for single-cell RNA-Seq based on a multinomial model. *Genome Biol.* **20,** 295 (2019).
18. Song, D., Li, K., Hemminger, Z., Wollman, R. & Li, J. J. scPNMF: sparse gene encoding of single cells to facilitate gene selection for targeted gene profiling. *Bioinformatics* **37,** i358–i366 (2021).
19. Vandenbon, A. & Diez, D. A clustering-independent method for finding differentially expressed genes in single-cell transcriptome data. *Nat. Commun.* **11,** 4318 (2020).
20. DeTomaso, D. & Yosef, N. Hotspot identifies informative gene modules across modalities of single-cell genomics. *Cell Syst* (2021). doi:10.1016/j.cels.2021.04.005
21. M Ascensión, A., Ibáñez-Solé, O., Inza, I., Izeta, A. & Araúzo-Bravo, M. J. Triku: a feature selection method based on nearest neighbors for single-cell data. *Gigascience* **11,** (2022).
22. Lambert, S. A., Jolma, A., Campitelli, L. F., Das, P. K., Yin, Y., Albu, M., Chen, X., Taipale, J., Hughes, T. R. & Weirauch, M. T. The Human Transcription Factors. *Cell* **172,** 650–665 (2018).
23. The Human Transcription Factors. at <https://humantfs.ccbr.utoronto.ca/index.php>
24. Lin, Y., Ghazanfar, S., Strbenac, D., Wang, A., Patrick, E., Lin, D. M., Speed, T., Yang, J. Y. H. & Yang, P.

Evaluating stably expressed genes in single cells. *Gigascience* **8,** (2019).

25. Luecken, M. D., Büttner, M., Chaichoompu, K., Danese, A., Interlandi, M., Mueller, M. F., Strobl, D. C., Zappia, L., Dugas, M., Colomé-Tatché, M. & Theis, F. J. Benchmarking atlas-level data integration in single-cell genomics. *Nat. Methods* (2021). doi:10.1038/s41592-021-01336-8
26. Pedregosa, F., Varoquaux, G., Gramfort, A., Michel, V., Thirion, B., Grisel, O., Blondel, M., Prettenhofer, P., Weiss, R., Dubourg, V., Vanderplas, J., Passos, A., Cournapeau, D., Brucher, M., Perrot, M. & Duchesnay, É. Scikit-learn: Machine Learning in Python. *J. Mach. Learn. Res.* **12,** 2825–2830 (2011).
27. Korsunsky, I., Millard, N., Fan, J., Slowikowski, K., Zhang, F., Wei, K., Baglaenko, Y., Brenner, M., Loh, P.-R. & Raychaudhuri, S. Fast, sensitive and accurate integration of single-cell data with Harmony. *Nat. Methods* (2019). doi:10.1038/s41592-019-0619-0
28. Büttner, M., Miao, Z., Wolf, F. A., Teichmann, S. A. & Theis, F. J. A test metric for assessing single-cell RNA-seq batch correction. *Nat. Methods* **16,** 43–49 (2019).
29. Butler, A., Hoffman, P., Smibert, P., Papalexi, E. & Satija, R. Integrating single-cell transcriptomic data across different conditions, technologies, and species. *Nat. Biotechnol.* (2018). doi:10.1038/nbt.4096
30. Lütge, A., Zyprych-Walczak, J., Brykczynska Kunzmann, U., Crowell, H. L., Calini, D., Malhotra, D., Soneson, C. & Robinson, M. D. CellMixS: quantifying and visualizing batch effects in single-cell RNA-seq data. *Life Sci Alliance* **4,** (2021).
31. Tirosh, I., Izar, B., Prakadan, S. M., Wadsworth, M. H., 2nd, Treacy, D., Trombetta, J. J., Rotem, A., Rodman, C., Lian, C., Murphy, G., Fallahi-Sichani, M., Dutton-Regester, K., Lin, J.-R., Cohen, O., Shah, P., Lu, D., Genshaft, A. S., Hughes, T. K., Ziegler, C. G. K., Kazer, S. W., Gaillard, A., Kolb, K. E., Villani, A.-C., Johannessen, C. M., Andreev, A. Y., Van Allen, E. M., Bertagnolli, M., Sorger, P. K., Sullivan, R. J., Flaherty, K. T., Frederick, D. T., Jané-Valbuena, J., Yoon, C. H., Rozenblatt-Rosen, O., Shalek, A. K., Regev, A. & Garraway, L. A. Dissecting the multicellular ecosystem of metastatic melanoma by single-cell RNA-seq. *Science* **352,** 189–196 (2016).
32. Smedley, D., Haider, S., Durinck, S., Pandini, L., Provero, P., Allen, J., Arnaiz, O., Awedh, M. H., Baldock, R., Barbiera, G., Bardou, P., Beck, T., Blake, A., Bonierbale, M., Brookes, A. J., Bucci, G., Buetti, I., Burge, S., Cabau, C., Carlson, J. W., Chelala, C., Chrysostomou, C., Cittaro, D., Collin, O., Cordova, R., Cutts, R. J.,

Dassi, E., Di Genova, A., Djari, A., Esposito, A., Estrella, H., Eyras, E., Fernandez-Banet, J., Forbes, S., Free, R. C., Fujisawa, T., Gadaleta, E., Garcia-Manteiga, J. M., Goodstein, D., Gray, K., Guerra-Assunção, J. A., Haggarty, B., Han, D.-J., Han, B. W., Harris, T., Harshbarger, J., Hastings, R. K., Hayes, R. D., Hoede, C., Hu, S., Hu, Z.-L., Hutchins, L., Kan, Z., Kawaji, H., Keliet, A., Kerhornou, A., Kim, S., Kinsella, R., Klopp, C., Kong, L., Lawson, D., Lazarevic, D., Lee, J.-H., Letellier, T., Li, C.-Y., Lio, P., Liu, C.-J., Luo, J., Maass, A., Mariette, J., Maurel, T., Merella, S., Mohamed, A. M., Moreews, F., Nabihoudine, I., Ndegwa, N., Noirot, C., Perez-Llamas, C., Primig, M., Quattrone, A., Quesneville, H., Rambaldi, D., Reecy, J., Riba, M., Rosanoff, S., Saddiq, A. A., Salas, E., Sallou, O., Shepherd, R., Simon, R., Sperling, L., Spooner, W., Staines, D. M., Steinbach, D., Stone, K., Stupka, E., Teague, J. W., Dayem Ullah, A. Z., Wang, J., Ware, D., Wong-Erasmus, M., Youens-Clark, K., Zadissa, A., Zhang, S.-J. & Kasprzyk, A. The BioMart community portal: an innovative alternative to large, centralized data repositories. *Nucleic Acids Res.* **43,** W589–98 (2015).

33. Durinck, S., Moreau, Y., Kasprzyk, A., Davis, S., De Moor, B., Brazma, A. & Huber, W. BioMart and Bioconductor: a powerful link between biological databases and microarray data analysis. *Bioinformatics* **21,** 3439–3440 (2005).

34. Maan, H., Zhang, L., Yu, C., Geuenich, M. J., Campbell, K. R. & Wang, B. Characterizing the impacts of dataset imbalance on single-cell data integration. *Nat. Biotechnol.* (2024). doi:10.1038/s41587-023-02097-9

35. Latecki, L. J., Lazarevic, A. & Pokrajac, D. Outlier Detection with Kernel Density Functions. in *Machine Learning and Data Mining in Pattern Recognition: 5th International Conference, MLDM 2007, Leipzig, Germany, July 18-20, 2007, Proceedings* (ed. Perner, P.) (Springer, 2007). at <https://play.google.com/store/books/details?id=pkxsCQAAQBAJ>

36. Kang, J. B., Nathan, A., Weinand, K., Zhang, F., Millard, N., Rumker, L., Moody, D. B., Korsunsky, I. & Raychaudhuri, S. Efficient and precise single-cell reference atlas mapping with Symphony. *Nat. Commun.* **12,** 5890 (2021).

37. Dann, E., Cujba, A.-M., Oliver, A. J., Meyer, K. B., Teichmann, S. A. & Marioni, J. C. Precise identification of cell states altered in disease using healthy single-cell references. *Nat. Genet.* (2023).

doi:10.1038/s41588-023-01523-7

38. Gupta, A., Tatbul, N., Marcus, R., Zhou, S., Lee, I. & Gottschlich, J. Class-Weighted Evaluation Metrics for Imbalanced Data Classification. *arXiv [cs.LG]* (2020). at <http://arxiv.org/abs/2010.05995>

39. Matthews, B. W. Comparison of the predicted and observed secondary structure of T4 phage lysozyme. *Biochim. Biophys. Acta* **405,** 442–451 (1975).

40. Yule, G. U. On the Methods of Measuring Association Between Two Attributes. *J. R. Stat. Soc.* **75,** 579–652 (1912).

41. Dann, E., Henderson, N. C., Teichmann, S. A., Morgan, M. D. & Marioni, J. C. Differential abundance testing on single-cell data using k-nearest neighbor graphs. *Nat. Biotechnol.* **40,** 245–253 (2022).

42. Luecken, M., Buttner, M., Danese, A., Interlandi, M., Müller, M., Strobl, D., Zappia, L., Dugas, M., Colomé-Tatché, M., Theis, F. & Chaichoompu, K. Benchmarking atlas-level data integration in single-cell genomics - integration task datasets. (2022). at <https://figshare.com/articles/dataset/Benchmarking_atlas-level_data_integration_in_single-cell_genomics_-_integration_task_datasets_Immune_and_pancreas_/12420968>

43. Lance, C., Luecken, M. D., Burkhardt, D. B., Cannoodt, R., Rautenstrauch, P., Laddach, A., Ubingazhibov, A., Cao, Z.-J., Deng, K., Khan, S., Liu, Q., Russkikh, N., Ryazantsev, G., Ohler, U., Data integration competition participants, N. 2021 M., Pisco, A. O., Bloom, J., Krishnaswamy, S. & Theis, F. J. Multimodal single cell data integration challenge: Results and lessons learned. in *Proceedings of the NeurIPS 2021 Competitions and Demonstrations Track* (eds. Kiela, D., Ciccone, M. & Caputo, B.) **176,** 162–176 (PMLR, 06--14 Dec 2022).

44. Luecken, M. D., Burkhardt, D. B., Cannoodt, R., Lance, C., Agrawal, A., Aliee, H., Chen, A. T., Deconinck, L., Detweiler, A. M., Granados, A. A., Huynh, S., Isacco, L., Kim, Y. J., Klein, D., de Kumar, B., Kuppasani, S., Lickert, H., McGeever, A., Mekonen, H., Melgarejo, J. C., Morri, M., Müller, M., Neff, N., Paul, S., Rieck, B., Schneider, K., Steelman, S., Sterr, M., Treacy, D. J., Tong, A., Villani, A.-C., Wang, G., Yan, J., Zhang, C., Pisco, A. O., Krishnaswamy, S., Theis, F. J. & Bloom, J. M. A sandbox for prediction and integration of DNA, RNA, and proteins in single cells. in *Thirty-fifth Conference on Neural Information Processing Systems Datasets and Benchmarks Track (Round 2)* (2022). at

<https://openreview.net/pdf?id=gN35BGa1Rt>

45. Clough, E., Barrett, T., Wilhite, S. E., Ledoux, P., Evangelista, C., Kim, I. F., Tomashevsky, M., Marshall, K. A., Phillippy, K. H., Sherman, P. M., Lee, H., Zhang, N., Serova, N., Wagner, L., Zalunin, V., Kochergin, A. & Soboleva, A. NCBI GEO: archive for gene expression and epigenomics data sets: 23-year update. *Nucleic Acids Res.* (2023). doi:10.1093/nar/gkad965
46. Popescu, D.-M., Botting, R. A., Stephenson, E., Green, K., Webb, S., Jardine, L., Calderbank, E. F., Polanski, K., Goh, I., Efremova, M., Acres, M., Maunder, D., Vegh, P., Gitton, Y., Park, J.-E., Vento-Tormo, R., Miao, Z., Dixon, D., Rowell, R., McDonald, D., Fletcher, J., Poyner, E., Reynolds, G., Mather, M., Moldovan, C., Mamanova, L., Greig, F., Young, M. D., Meyer, K. B., Lisgo, S., Bacardit, J., Fuller, A., Millar, B., Innes, B., Lindsay, S., Stubbington, M. J. T., Kowalczyk, M. S., Li, B., Ashenberg, O., Tabaka, M., Dionne, D., Tickle, T. L., Slyper, M., Rozenblatt-Rosen, O., Filby, A., Carey, P., Villani, A.-C., Roy, A., Regev, A., Chédotal, A., Roberts, I., Göttgens, B., Behjati, S., Laurenti, E., Teichmann, S. A. & Haniffa, M. Decoding human fetal liver haematopoiesis. *Nature* **574,** 365–371 (2019).
47. Haniffa Lab. Fetal Liver. *CellAtlas.io* at <https://app.cellatlas.io/fetal-liver/>
48. Reed, A. D., Pensa, S., Steif, A., Stenning, J., Kunz, D. J., Porter, L. J., Hua, K., He, P., Twigger, A.-J., Siu, A. J. Q., Kania, K., Barrow-McGee, R., Goulding, I., Gomm, J. J., Speirs, V., Jones, J. L., Marioni, J. C. & Khaled, W. T. A single-cell atlas enables mapping of homeostatic cellular shifts in the adult human breast. *Nat. Genet.* **56,** 652–662 (2024).
49. Reed, A. D., Pensa, S., Steif, A., Stenning, J., Kunz, D. J., He, P., Twigger, A.-J., Kania, K., Barrow-McGee, R., Goulding, I., Gomm, J. J., Jones, L., Marioni, J. C. & Khaled, W. T. A Human Breast Cell Atlas Mapping the Homeostatic Cellular Shifts in the Adult Breast. *bioRxiv* 2023.04.21.537845 (2023). doi:10.1101/2023.04.21.537845
50. Chan Zuckerberg Initiative. CELLxGene Data Portal. *Chan Zuckerberg CELLxGENE Discover* at <https://cellxgene.cziscience.com/>
51. Swamy, V. S., Fufa, T. D., Hufnagel, R. B. & McGaughey, D. M. Building the mega single-cell transcriptome ocular meta-atlas. *Gigascience* **10,** (2021).
52. McGaughey, D. & National Eye Institute. Data. *plae: PLatform for Analysis of scEiad* at

<https://plae.nei.nih.gov/>

53. Yu, Q., Kilik, U., Holloway, E. M., Tsai, Y.-H., Harmel, C., Wu, A., Wu, J. H., Czerwinski, M., Childs, C. J., He, Z., Capeling, M. M., Huang, S., Glass, I. A., Higgins, P. D. R., Treutlein, B., Spence, J. R. & Camp, J. G. Charting human development using a multi-endodermal organ atlas and organoid models. *Cell* **184,** 3281–3298.e22 (2021).
54. Yu, Q., Kilik, U., Holloway, E. M., Tsai, Y.-H., Harmel, C., Wu, A., Wu, J. H., Czerwinski, M., Childs, C., He, Z., Capeling, M. M., Huang, S., Glass, I., Higgins, P. D. R., Treutlein, B., Spence, J. R. & Camp, J. G. Charting human development using a multi-organ atlas and organoid models. (2021). at <https://data.mendeley.com/datasets/x53tts3zfr/2>
55. Sikkema, L., Ramírez-Suástegui, C., Strobl, D. C., Gillett, T. E., Zappia, L., Madissoon, E., Markov, N. S., Zaragosi, L.-E., Ji, Y., Ansari, M., Arguel, M.-J., Apperloo, L., Banchero, M., Bécavin, C., Berg, M., Chichelnitskiy, E., Chung, M.-I., Collin, A., Gay, A. C. A., Gote-Schniering, J., Hooshiar Kashani, B., Inecik, K., Jain, M., Kapellos, T. S., Kole, T. M., Leroy, S., Mayr, C. H., Oliver, A. J., von Papen, M., Peter, L., Taylor, C. J., Walzthoeni, T., Xu, C., Bui, L. T., De Donno, C., Dony, L., Faiz, A., Guo, M., Gutierrez, A. J., Heumos, L., Huang, N., Ibarra, I. L., Jackson, N. D., Kadur Lakshminarasimha Murthy, P., Lotfollahi, M., Tabib, T., Talavera-López, C., Travaglini, K. J., Wilbrey-Clark, A., Worlock, K. B., Yoshida, M., Lung Biological Network Consortium, van den Berge, M., Bossé, Y., Desai, T. J., Eickelberg, O., Kaminski, N., Krasnow, M. A., Lafyatis, R., Nikolic, M. Z., Powell, J. E., Rajagopal, J., Rojas, M., Rozenblatt-Rosen, O., Seibold, M. A., Sheppard, D., Shepherd, D. P., Sin, D. D., Timens, W., Tsankov, A. M., Whitsett, J., Xu, Y., Banovich, N. E., Barbry, P., Duong, T. E., Falk, C. S., Meyer, K. B., Kropski, J. A., Pe'er, D., Schiller, H. B., Tata, P. R., Schultze, J. L., Teichmann, S. A., Misharin, A. V., Nawijn, M. C., Luecken, M. D. & Theis, F. J. An integrated cell atlas of the lung in health and disease. *Nat. Med.* **29,** 1563–1577 (2023).
56. Zappia, L., Phipson, B. & Oshlack, A. Splatter: simulation of single-cell RNA sequencing data. *Genome Biol.* **18,** 174 (2017).
57. R Core Team. R: A Language and Environment for Statistical Computing. Preprint at https://www.R-project.org/ (2021)
58. Huber, W., Carey, V. J., Gentleman, R., Anders, S., Carlson, M., Carvalho, B. S., Bravo, H. C., Davis, S.,

Gatto, L., Girke, T., Gottardo, R., Hahne, F., Hansen, K. D., Irizarry, R. A., Lawrence, M., Love, M. I., MacDonald, J., Obenchain, V., Oleś, A. K., Pagès, H., Reyes, A., Shannon, P., Smyth, G. K., Tenenbaum, D., Waldron, L. & Morgan, M. Orchestrating high-throughput genomic analysis with Bioconductor. *Nat. Methods* **12,** 115–121 (2015).

59. Virshup, I., Rybakov, S., Theis, F. J., Angerer, P. & Wolf, F. A. anndata: Access and store annotated data matrices. *J. Open Source Softw.* **9,** 4371 (2024).
60. Lopez, R., Regier, J., Cole, M. B., Jordan, M. I. & Yosef, N. Deep generative modeling for single-cell transcriptomics. *Nat. Methods* **15,** 1053–1058 (2018).
61. Gayoso, A., Lopez, R., Xing, G., Boyeau, P., Valiollah Pour Amiri, V., Hong, J., Wu, K., Jayasuriya, M., Mehlman, E., Langevin, M., Liu, Y., Samaran, J., Misrachi, G., Nazaret, A., Clivio, O., Xu, C., Ashuach, T., Gabitto, M., Lotfollahi, M., Svensson, V., da Veiga Beltrame, E., Kleshchevnikov, V., Talavera-López, C., Pachter, L., Theis, F. J., Streets, A., Jordan, M. I., Regier, J. & Yosef, N. A Python library for probabilistic analysis of single-cell omics data. *Nat. Biotechnol.* (2022). doi:10.1038/s41587-021-01206-w
62. Lotfollahi, M., Naghipourfar, M., Luecken, M. D., Khajavi, M., Büttner, M., Wagenstetter, M., Avsec, Ž., Gayoso, A., Yosef, N., Interlandi, M., Rybakov, S., Misharin, A. V. & Theis, F. J. Mapping single-cell data to reference atlases by transfer learning. *Nat. Biotechnol.* (2021). doi:10.1038/s41587-021-01001-7
63. Xu, C., Lopez, R., Mehlman, E., Regier, J., Jordan, M. I. & Yosef, N. Probabilistic harmonization and annotation of single-cell transcriptomics data with deep generative models. *Mol. Syst. Biol.* **17,** e9620 (2021).
64. Slowikowski, K., @slowkow, @johnarevalo, @pinin4fjords & @bli. *harmonypy: Integrate multiple high-dimensional datasets with fuzzy k-means and locally linear adjustments*. (GitHub). at <https://github.com/slowkow/harmonypy>
65. Petrova, K., @potulabe, @serjisa & @maarten-devries. *symphonypy: Port of symphony algorithm of single-cell reference atlas mapping to Python*. (GitHub). at <https://github.com/potulabe/symphonypy>
66. Heumos, L., Schaar, A. C., Lance, C., Litinetskaya, A., Drost, F., Zappia, L., Lücken, M. D., Strobl, D. C., Henao, J., Curion, F., Single-cell Best Practices Consortium, Schiller, H. B. & Theis, F. J. Best practices for

single-cell analysis across modalities. *Nat. Rev. Genet.* 1–23 (2023). doi:10.1038/s41576-023-00586-w

67. Wickham, H. *ggplot2: Elegant Graphics for Data Analysis*. (Springer New York, 2010).

68. Wickham, H., Averick, M., Bryan, J., Chang, W., McGowan, L., François, R., Grolemund, G., Hayes, A., Henry, L., Hester, J., Kuhn, M., Pedersen, T., Miller, E., Bache, S., Müller, K., Ooms, J., Robinson, D., Seidel, D., Spinu, V., Takahashi, K., Vaughan, D., Wilke, C., Woo, K. & Yutani, H. Welcome to the Tidyverse. *JOSS* **4,** 1686 (2019).
