# Supplementary material for: Feature selection methods affect the performance of scRNA-seq data integration and querying
Source: Nat Methods. 2025 Mar 13;22(4):834–44. doi: 10.1038/s41592-025-02624-3 (PMC11978513; doi:10.1038/s41592-025-02624-3)
Supplement: Supplementary file 2 — Reporting Summary [file 41592_2025_2624_MOESM2_ESM.pdf]

nature portfolio

Corresponding author(s): Fabian Theis

Last updated by author(s): Sep 29, 2024

Reporting Summary

Nature Portfolio wishes to improve the reproducibility of the work that we publish. This form provides structure for consistency and transparency in reporting. For further information on Nature Portfolio policies, see our Editorial Policies and the Editorial Policy Checklist.

Statistics

For all statistical analyses, confirm that the following items are present in the figure legend, table legend, main text, or Methods section.

n/a	Confirmed	
☒	☐	The exact sample size (*n*) for each experimental group/condition, given as a discrete number and unit of measurement
☒	☐	A statement on whether measurements were taken from distinct samples or whether the same sample was measured repeatedly
☒	☐	The statistical test(s) used AND whether they are one- or two-sided<br>*Only common tests should be described solely by name; describe more complex techniques in the Methods section.*
☒	☐	A description of all covariates tested
☒	☐	A description of any assumptions or corrections, such as tests of normality and adjustment for multiple comparisons
☒	☐	A full description of the statistical parameters including central tendency (e.g. means) or other basic estimates (e.g. regression coefficient) AND variation (e.g. standard deviation) or associated estimates of uncertainty (e.g. confidence intervals)
☒	☐	For null hypothesis testing, the test statistic (e.g. *F*, *t*, *r*) with confidence intervals, effect sizes, degrees of freedom and *P* value noted<br>*Give P values as exact values whenever suitable.*
☒	☐	For Bayesian analysis, information on the choice of priors and Markov chain Monte Carlo settings
☒	☐	For hierarchical and complex designs, identification of the appropriate level for tests and full reporting of outcomes
☒	☐	Estimates of effect sizes (e.g. Cohen's *d*, Pearson's *r*), indicating how they were calculated

Our web collection on statistics for biologists contains articles on many of the points above.

Software and code

Policy information about availability of computer code

Data collection

All code associated with this study is available on GitHub (https://github.com/theislab/atlas-feature-selection-benchmark/analysis) and archived on Zenodo (https://doi.org/10.5281/ZENODO.13995812), including scripts for downloading datasets from public repositories provided by the original authors, running methods and calculating metrics, the Nextflow pipeline and associated environment and configuration files. The code for analysing the benchmark results, including the production of final figures, is also available in this repository.

Data analysis

Analysis of benchmark results was performed in R and Python using standard packages. All code is provided in the code repository on GitHub (https://github.com/theislab/atlas-feature-selection-benchmark/analysis) and archived on Zenodo (https://doi.org/10.5281/ZENODO.13995812). Rendered analysis reports including the package versions used are available from figshare (https://doi.org/10.6084/M9.FIGSHARE.C.7521966).

Key package versions:

anndata (v0.8.0)
anticor (v0.1.8)
balanced_clustering (commit: a2ae3a4d)
batchelor (v1.14.0)
biomaRt (v2.54.0)
CellMixS (v1.14.0)
cellxgene-census (v1.0.1)
DUBStepR (commit 76aa3948)
ggplot2 (v3.5.0)

harmonypy (v0.0.9)
hotspot (v1.0.0)
kBET (commit a10ffeaa)
m3drop (v1.24.0)
milopy (commit be1a6cc8)
numpy (v1.22.4)
pandas (v1.4.3)
patchwork (v1.2.0)
Python (v3.9.13)
R (v4.2.2)
reticulate (v1.26)
scanpy (v1.9.1)
scIB (v1.1.4)
scikit-learn (v1.1.2)
scipy (v1.9.0)
scMerge (v1.1.4.0)
scPNMF (commit 47d5b10c)
scran (v1.26.0)
scry (v1.10.0)
scTransform (v0.3.5)
scuttle (v1.8.0)
scvi-tools (v0.17.1)
Seurat (v4.3.0)
SeuratObject (v4.1.3)
SingleCellExperiment (v1.20.0)
singleCellHaystack (v0.3.4)
splatter (v1.25.1)
symphonypy (0.2.1)
tidyverse (v2.0.0)
triku (v2.1.4)
zellkonverter (v1.8.0)

For manuscripts utilizing custom algorithms or software that are central to the research but not yet described in published literature, software must be made available to editors and reviewers. We strongly encourage code deposition in a community repository (e.g. GitHub). See the Nature Portfolio guidelines for submitting code & software for further information.

Data

Policy information about availability of data

All manuscripts must include a data availability statement. This statement should provide the following information, where applicable:

- Accession codes, unique identifiers, or web links for publicly available datasets
- A description of any restrictions on data availability
- For clinical datasets or third party data, please ensure that the statement adheres to our policy

All real scRNA-seq datasets were downloaded from public repositories provided by the original authors as described in the methods (scIB Pancreas: figshare(https://figshare.com/articles/dataset/Benchmarking_atlas-level_data_integration_in_single-cell_genomics_-_integration_task_datasets_Immune_and_pancreas_/12420968), NeurIPS: GEO (GSE194122), Fetal liver: CellAtlas.io, Reed Breast: CELLxGENE Discover (Dataset ID: 0ba636a1-4754-4786-a8be-7ab3cf760fd6, Census version: 2023-07-25), scEiaD: PLatform for Analysis of scEiad website (https://plae.nei.nih.gov/), Human endoderm: Mendelay Data (https://data.mendeley.com/datasets/x53tts3zfr/2), HLCA: CELLxGENE Discover (Dataset ID: 066943a2-fdac-4b29-b348-40cede398e4e, Census version: 2023-07-25). Raw and prepared dataset files, selected feature sets, metric scores and rendered analysis reports from this benchmark are available from figshare (https://doi.org/10.6084/M9.FIGSHARE.C.7521966).

Research involving human participants, their data, or biological material

Policy information about studies with human participants or human data. See also policy information about sex, gender (identity/presentation), and sexual orientation and race, ethnicity and racism.

Reporting on sex and gender	N/A
Reporting on race, ethnicity, or other socially relevant groupings	N/A
Population characteristics	N/A
Recruitment	N/A
Ethics oversight	N/A

Note that full information on the approval of the study protocol must also be provided in the manuscript.

Field-specific reporting

Please select the one below that is the best fit for your research. If you are not sure, read the appropriate sections before making your selection.

☒ Life sciences ☐ Behavioural & social sciences ☐ Ecological, evolutionary & environmental sciences

For a reference copy of the document with all sections, see nature.com/documents/nr-reporting-summary-flat.pdf

Life sciences study design

All studies must disclose on these points even when the disclosure is negative.

Sample size	The number of datasets in the study was chosen to cover a representative sample of scenarios and tissues as discussed and approved by reviewers in the Stage 1 review. The number of samples in each dataset was as provided by the original authors.
Data exclusions	For some datasets, a subset of the data was used. Either to select a relevant part of the data (such as a single tissue/species) or to for technical reasons (i.e. labels with too few cells). Any subsetting of datasets is described in that dataset's section in the manuscript.
Replication	Due to computational limitations the main benchmark run was performed a single time. We did not attempt to replicate the results through an independent run of the benchmarking pipeline.
Randomization	All correlations shown in the manuscript were performed between pairs of variables without considering other covariates. We did not perform any statistical testing for which covariates should be considered
Blinding	All running and scoring of methods was performed by an automated workflow.

Reporting for specific materials, systems and methods

We require information from authors about some types of materials, experimental systems and methods used in many studies. Here, indicate whether each material, system or method listed is relevant to your study. If you are not sure if a list item applies to your research, read the appropriate section before selecting a response.

Materials & experimental systems

n/a	Involved in the study
☒	☐ Antibodies
☒	☐ Eukaryotic cell lines
☒	☐ Palaeontology and archaeology
☒	☐ Animals and other organisms
☒	☐ Clinical data
☒	☐ Dual use research of concern
☒	☐ Plants

Methods

n/a	Involved in the study
☒	☐ ChIP-seq
☒	☐ Flow cytometry
☒	☐ MRI-based neuroimaging

Plants

Seed stocks	*Report on the source of all seed stocks or other plant material used. If applicable, state the seed stock centre and catalogue number. If plant specimens were collected from the field, describe the collection location, date and sampling procedures.*
Novel plant genotypes	*Describe the methods by which all novel plant genotypes were produced. This includes those generated by transgenic approaches, gene editing, chemical/radiation-based mutagenesis and hybridization. For transgenic lines, describe the transformation method, the number of independent lines analyzed and the generation upon which experiments were performed. For gene-edited lines, describe the editor used, the endogenous sequence targeted for editing, the targeting guide RNA sequence (if applicable) and how the editor was applied.*
Authentication	*Describe any authentication procedures for each seed stock used or novel genotype generated. Describe any experiments used to assess the effect of a mutation and, where applicable, how potential secondary effects (e.g. second site T-DNA insertions, mosiacism, off-target gene editing) were examined.*
